# Author Correction: *Culex pipiens* crossing type diversity is governed by an amplified and polymorphic operon of *Wolbachia*

**DOI:** 10.1038/s41467-018-03799-4

**Published:** 2018-04-11

**Authors:** Manon Bonneau, Celestine Atyame, Marwa Beji, Fabienne Justy, Martin Cohen-Gonsaud, Mathieu Sicard, Mylène Weill

**Affiliations:** 1 Institut des Sciences de l’Evolution de Montpellier (ISEM), UMR CNRS-IRD-EPHE-Université de Montpellier, Place Eugène Bataillon, 34095 Montpellier, France; 2 Processus Infectieux en Milieu Insulaire Tropical (PIMIT), UMR CNRS-INSERM-IRD-Université de La Réunion, Sainte-Clotilde, 97490 Ile de La Réunion, France; 3 Institut Pasteur Tunis, Laboratory of Epidemiology and Veterinary Microbiology, University of Tunis El Manar, 1068 Tunis, Tunisia; 4 Centre de Biochimie Structurale (CBS), UMR CNRS-INSERM-Université de Montpellier, 29 rue de Navacelles, 34090 Montpellier, France

Correction to: *Nature Communications* 10.1038/s41467-017-02749-w, published online 22 January 2018

In the originally published HTML and PDF versions of this Article, gel images in Figs. 7c and 8c were not prepared as per the *Nature* journal policy. These figure panels have now been corrected in both the PDF and HTML versions of the Article.

In Fig. 7c, the lane labelled ‘Ha’ was inappropriately duplicated to represent the lane labelled ‘Ich13’. The corrected version of Fig. 7c includes PCR-RFLP on DNA from the Ichkeul 13 line, which had been run on a separate gel. The original unprocessed gel images are provided in Supplementary Figure 1 associated with this correction, with the relevant corresponding bands denoted. A repeat experiment of the PCR-RFLP test is also presented as Supplementary Figure 2.

In Fig. 8c, the image was assembled from two separate gels without clear demarcation. The corrected Fig. 8c clearly separates lanes from the two gels, and the original unprocessed gel images are provided in the Supplementary Information associated with this correction.

These corrections do not alter the original meaning of the experiments, their results, their interpretation, or the conclusions of the paper. We apologize for any confusion this may have caused to the readers of *Nature Communications*.

## Electronic supplementary material

Supplementary Information

