# Supplementary material for: Culex pipiens crossing type diversity is governed by an amplified and polymorphic operon of Wolbachia
Source: Nat Commun. 2018 Jan 22;9:319. doi: 10.1038/s41467-017-02749-w (PMC5778026; doi:10.1038/s41467-017-02749-w)
Supplement: Supplementary file 1 — Supplementary Information [file 41467_2017_2749_MOESM1_ESM.pdf]

Culex pipiens crossing type diversity is governed by an amplified and polymorphic operon of *Wolbachia*

Bonneau *et al*.

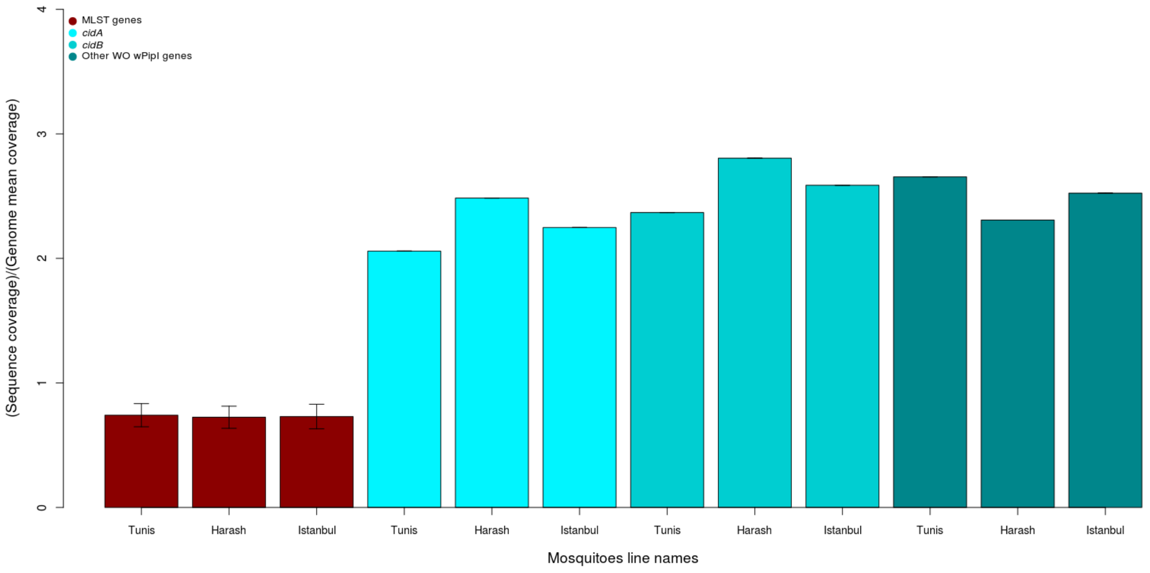

Supplementary Figure 1 | Mapping depth coverages of *cidA* and *cidB* suggest an amplification of these genes in *w*Pip genomes. Comparison of the coverage depth obtained from the mapping of the Illumina reads of *w*Pip hosted by Tunis, Istanbul and Harash *C. pipiens* isofemale lines on the reference genome *w*Pip_Pel between *cidA*, *cidB*, WO *w*Pip prophage I genes (without *cidA* and *cidB*) and 14 housekeeping genes (gatB, coxA, hcpA, fbpA, ftsZ, aspC; atpD, sucB, pdhB, 16s, wsp, groEl, dnaA, dltA) that were predicted to be in single copy in the genome of all *Wolbachia*. Coverage for each line has been normalized by the mean coverage of this line on the reference genome. Error bars represent the standard error on the mean of the average coverage of the 14 housekeeping genes.

```
CidA_I(α/1)     MPTQKELRDTMSKKLQEAIKHPDPAVVAGRKSAIKRWVGVLQDNFMEHIKYFKGDKLKFL
CidA_I(γ/1)     MPTQKELRDTMSKKLQEAIKHPDPAVVAGRKSAIKRWVGVLQDNFMEHIKYFKGDKLKFL
CidA_I(γ/2)     MPTQKELRDTMSKKLQEAIKHPDPAVVAGRKSAIKRWVGVLQDNFMEHIKYFKGDKLKFL
CidA_I(β/2)     MPTQKELRDTMSKKLQEAIKHPDPAVVAGRKSAIKRWVGVLQDNFMEHIKYFKGDKLKFL
CidA_II(α/1)    MPTQKELRDTMSKKLQEAIKHPDPAVVAGRKSAIKRWVGVLQDNFMEHIKYFKGDKLKFL
CidA_II(α/2)    MPTQKELRDTMSKKLQEAIKHPDPAVVAGRKSAIKRWVGVLQDNFMEHIKYFKGDKLKFL
CidA_II(β/2)    MPTQKELRDTMSKKLQEAIKHPDPAVVAGRKSAIKRWVGVLQDNFMEHIKYFKGDKLKFL
CidA_III(α/1)   MPTQKELRDTMSKKLQEAIKHPDPAVVAGRKSAIKRWVGVLQDNFMEHIKYFKGDKLKFL
CidA_III(β/2)   MPTQKELRDTMSKKLQEAIKHPDPAVVAGRKSAIKRWVGVLQDNFMEHIKYFKGDKLKFL
CidA_III(β/1)   MPTQKELRDTMSKKLQEAIKHPDPAVVAGRKSAIKRWVGVLQDNFMEHIKYFKGDKLKFL
CidA_III(β/3)   MPTQKELRDTMSKKLQEAIKHPDPAVVAGRKSAIKRWVGVLQDNFMEHIKYFKGDKLKFL
CidA_IV(α/1)    MPTQKELRDTMSKKLQEAIKHPDPAVVAGRKSAIKRWVGVLQDNFMEHIKYFKGDKLKFL
CidA_IV(α/2)    MPTQKELRDTMSKKLQEAIKHPDPAVVAGRKSAIKRWVGVLQDNFMEHIKYFKGDKLKFL
CidA_IV(γ/1)    MPTQKELRDTMSKKLQEAIKHPDPAVVAGRKSAIKRWVGVLQDNFMEHIKYFKGDKLKFL
CidA_IV(γ/2)    MPTQKELRDTMSKKLQEAIKHPDPAVVAGRKSAIKRWVGVLQDNFMEHIKYFKGDKLKFL
CidA_IV(δ/1)    MPTQKELRDTMSKKLQEAIKHPDPAVVAGRKSAIKRWVGVLQDNFMEHIKYFKGDKLKFL
CidA_IV(δ/2)    MPTQKELRDTMSKKLQEAIKHPDPAVVAGRKSAIKRWVGVLQDNFMEHIKYFKGDKLKFL

CidA_I(α/1)     HNVFQDEGCWSGVRLDNAALGQRFTEEKIGGIDNPLRKYEMACSYCVVDKIHPLFQKAFE
CidA_I(γ/1)     HNVFQDEGCWSGVRLDNAALGQRFTEEKIGGIDNPLRKYEMACSYCVVDKIHPLFQKRFE
CidA_I(γ/2)     HNVFQDEGCWSGVRLDNAALGQRFTEEKIGGIDNPLRKYEMACSYCVVDKIHPLFQKRFE
CidA_I(β/2)     HNVFQDEGCWSGVRLDNAALGQRFTEEKIGGIDNPLRKYEMACSYCVVDKIHPLFQKAFE
CidA_II(α/1)    HNVFQDEGCWSGVRLDNAALGQRFTEEKIGGIDNPLRKYEMACSYCVVDKIHPLFQKAFE
CidA_II(α/2)    HNVFQDEGCWSGVRLDNAALGQRFTEEKIGGIDNPLRKYEMACSYCVVDKIHPLFQKAFE
CidA_II(β/2)    HNVFQDEGCWSGVRLDNAALGQRFTEEKIGGIDNPLRKYEMACSYCVVDKIHPLFQKRFE
CidA_III(α/1)   HNVFQDEGCWSGVRLDNAALGQRFTEEKIGGIDNPLRKYEMACSYCVVDKIHPLFQKRFE
CidA_III(β/2)   HNVFQDEGCWSGVRLDNAALGQRFTEEKIGGIDNPLRKYEMACSYCVVDKIHPLFQKAFE
CidA_III(β/1)   HNVFQDEGCWSGVRLDNAALGQRFTEEKIGGIDNPLRKYEMACSYCVVDKIHPLFQKAFE
CidA_III(β/3)   HNVFQDEGCWSGVRLDNAALGQRFTEEKIGGIDNPLRKYEMACSYCVVDKIHPLFQKAFE
CidA_IV(α/1)    HNVFQDEGCWSGVRLDNAALGQRFTEEKIGGIDNPLRKYEMACSYCVVDKIHPLFQKAFE
CidA_IV(α/2)    HNVFQDEGCWSGVRLDNAALGQRFTEEKIGGIDNPLRKYEMACSYCVVDKIHPLFQKAFE
CidA_IV(γ/1)    HNVFQDEGCWSGVRLDNAALGQRFTEEKIGGIDNPLRKYEMACSYCVVDKIHPLFQKRFE
CidA_IV(γ/2)    HNVFQDEGCWSGVRLDNAALGQRFTEEKIGGIDNPLRKYEMACSYCVVDKIHPLFQKRFE
CidA_IV(δ/1)    HNVFQDEGCWSGVRLDNAALGQRFTEEKIGGIDNPLRKYEMACSYCVVDKIHPLFQKRFE
CidA_IV(δ/2)    HNVFQDEGCWSGVRLDNAALGQRFTEEKIGGIDNPLRKYEMACSYCVVDKIHPLFQKRFE

CidA_I(α/1)     SYKNGFSSDELDSRGNPITDEYIRNSLLGGIRRKGPVFDFWIDRESGELKKYDAVEGFDS
CidA_I(γ/1)     SYRNKFPPGAFDGKTETEFGKYVRNSLLDSIKRKGPVFDFWIDRESGELKKYDAVEGFDS
CidA_I(γ/2)     SYRNKFPPGAFDGKTETEFGKYVRNSLLDSIKRKGPVFDFWIDRESGELKKYDAVEGFDS
CidA_I(β/2)     SYKNGFSSDELDSRGNPITDEYIRNSLLGGIRRKGPVFDFWIDRESGELKKYDAVEGFDS
CidA_II(α/1)    SYKNGFSSDELDSRGNPITDEYIRNSLLGGIRRKGPVFDFWIDRESGELKKYDAVEGFDS
CidA_II(α/2)    SYKNGFSSDELDSRGNPITDEYIRNSLLGGIRRKGPVFDFWIDRESGELKKYDAVEGFDS
CidA_II(β/2)    SYRNKFPPGAFDGKTETEFGKYVRNSLLDSIKRKGPVFDFWIDRESGELKKYDAVEGFDS
CidA_III(α/1)   SYRNKFPPGAFDGKTETEFGKYVRNSLLDSIKRKGPVFDFWIDRESGELKKYDAVEGFDS
CidA_III(β/2)   SYKNGFSSDELDSRGNPITDEYIRNSLLGGIRRKGPVFDFWIDRESGELKKYDAVEGFDS
CidA_III(β/1)   SYKNGFSSDELDSRGNPITDEYIRNSLLGGIRRKGPVFDFWIDRESGELKKYDAVEGFDS
CidA_III(β/3)   SYKNGFSSDELDSRGNPITDEYIRNSLLGGIRRKGPVFDFWIDRESGELKKYDAVEGFDS
CidA_IV(α/1)    SYKNGFSSDELDSRGNPITDEYIRNSLLGGIRRKGPVFDFWIDRESGELKKYDAVEGFDS
CidA_IV(α/2)    SYKNGFSSDELDSRGNPITDEYIRNSLLGGIRRKGPVFDFWIDRESGELKKYDAVEGFDS
CidA_IV(γ/1)    SYRNKFPPGAFDGKTETEFGKYVRNSLLDSIKRKGPVFDFWIDRESGELKKYDAVEGFDS
CidA_IV(γ/2)    SYRNKFPPGAFDGKTETEFGKYVRNSLLDSIKRKGPVFDFWIDRESGELKKYDAVEGFDS
CidA_IV(δ/1)    SYRNKFPPGAFDSRGNPITDEYIRNSLLGGIRRKGPVFDFWIDRESGELKKYDAVEGFDS
CidA_IV(δ/2)    SYRNKFPPGAFDSRGNPITDEYIRNSLLGGIRRKGPVFDFWIDRESGELKKYDAVEGFDS
```

```
CidA_I(α/1)      AVKFKWSEGVEYFYNHLKEEDKEKKLTEAILALSRVQSVEKDAPILDFCVNKIVDKDTLL
CidA_I(γ/1)      AVKFKWSEGVEYFYNHLKEEDKEKKLTEAILALSRVQSVEKDAPILDFCVNKIVDKDTLL
CidA_I(γ/2)      AVKFKWSEGVEYFYNHLKEEDKEKKLTEAILALSRVQSVEKDAPILDFCVNKIVDKDTLL
CidA_I(β/2)      AVKFKWSEGVEYFYNHLKEEDKEKKLTEAILALSRVQSVEKDAPILDFCVNKIVDKDTLL
CidA_II(α/1)     AVKLKWSEGVEYFYNHLKEEDKEKKLTEAILALSRVQSVEKDAPILDFCVNKIVDKDTLL
CidA_II(α/2)     AVKLKWSEGVEYFYNHLKEEDKEKKLTEAILALSRVQSVEKDAPILDFCVNKIVDKDTLL
CidA_II(β/2)     AVKLKWSEGVEYFYNHLKEEDKEKKLTEAILALSRVQSVEKDAPILDFCVNKIVDKDTLL
CidA_III(α/1)    AVKLKWSEGVEYFYNHLKEEDKEKKLTEAILALSRVQSVEKDAPILDFCVNKIVDKDTLL
CidA_III(β/2)    AVKLKWSEGVEYFYNHLKEEDKEKKLTEAILALSRVQSVEKDAPILDFCVNKIVDKDTLL
CidA_III(β/1)    AVKLKWSEGVEYFYNHLKEEDKEKKLTEAILALSRVQSVEKDAPILDFCVNKIVDKDTLL
CidA_III(β/3)    AVKLKWSEGVEYFYNHLKEEDKEKKLTEAILALSRVQSVEKDAPILDFCVNKIVDKDTLL
CidA_IV(α/1)     AVKLKWSEGVEYFYNHLKEEDKEKKLTEAILALSRVQSVEKDAPILDFCVNKIVDKDTLL
CidA_IV(α/2)     AVKLKWSEGVEYFYNHLKEEDKEKKLTEAILALSRVQSVEKDAPILDFCVNKIVDKDTLL
CidA_IV(γ/1)     AVKLKWSEGVEYFYNHLKEEDKEKKLTEAILALSRVQSVEKDAPILDFCVNKIVDKDTLL
CidA_IV(γ/2)     AVKLKWSEGVEYFYNHLKEEDKEKKLTEAILALSRVQSVEKDAPILDFCVNKIVDKDTLL
CidA_IV(δ/1)     AVKLKWSEGVEYFYNHLKEEDKEKKLTEAILALSRVQSVEKDAPILDFCVNKIVDKDTLL
CidA_IV(δ/2)     AVKLKWSEGVEYFYNHLKEEDKEKKLTEAILALSRVQSVEKDAPILDFCVNKIVDKDTLL
```

```
CidA_I(α/1)      QKLSQKDKGVYSLFVELIESCFFDTVHDLVQCWCYKEVSAGGDHSEKIFSQRDYELFLSS
CidA_I(γ/1)      QKLSQKDKGVYSLFVELIESCFFDTVHDLVQCWCYKEVSAGGDHSEKIFSQRDYELFLSS
CidA_I(γ/2)      QKLSQKDKGVYSLFVELIESCFFDTVHDLVQCWCYKEVSAGGDHSEKIFSQRDYELFLSS
CidA_I(β/2)      QKLSQKDKGVYSLFAELIESCFFDTVHDLVQCWCYKEVSAGGDHSEKIFSQRDYELFLSS
CidA_II(α/1)     QKLSQKDKGVYSLFAELIESCFFDTVHDLVQCWCYKEVSAGGDHSEKIFSQRDYELFLSS
CidA_II(α/2)     QKLSQKDKGVYSLFAELIESCFFDTVHDLVQCWCYKEVSARGDCSDKIFSQRDYELFLSS
CidA_II(β/2)     QKLSQKDKGVYSLFAELIESCFFDTVHDLVQCWCYKEVSARGDCSDKIFSQRDYELFLSS
CidA_III(α/1)    QKLSQKDKGVYSLFAELIESCFFDTVHDLVQCWCYKEVSAGGDHSEKIFSQRDYELFLSS
CidA_III(β/2)    QKLSQKDKGVYSLFAELIESCFFDTVHDLVQCWCYKEVSAGGDHSEKIFSQRDYELFLSS
CidA_III(β/1)    QKLSQKDKGVYSLFAELIESCFFDTVHDLVQCWCYKEVSAGGDHSEKIFSQRDYELFLSS
CidA_III(β/3)    QKLSQKDKGVYSLFAELIESCFFDTVHDLVQCWCYKEVSAGGDHSEKIFSQRDYELFLSS
CidA_IV(α/1)     QKLSQKDKGVYSLFAELIESCFFDTVHDLVQCWCYKEVSARGDCSDKIFSQRDYELFLSS
CidA_IV(α/2)     QKLSQKDKGVYSLFAELIESCFFDTVHDLVQCWCYKEVSARGDCSDKIFSQRDYELFLSS
CidA_IV(γ/1)     QKLSQKDKGVYSLFAELIESCFFDTVHDLVQCWCYKEVSARGDCSDKIFSQRDYELFLSS
CidA_IV(γ/2)     QKLSQKDKGVYSLFAELIESCFFDTVHDLVQCWCYKEVSARGDCSDKIFSQRDYELFLSS
CidA_IV(δ/1)     QKLSQKDKGVYSLFAELIESCFFDTVHDLVQCWCYKEVSARGDCSDKIFSQRDYELFLSS
CidA_IV(δ/2)     QKLSQKDKGVYSLFAELIESCFFDTVHDLVQCWCYKEVSARGDCSDKIFSQRDYELFLSS
```

```
CidA_I(α/1)      LSDTMLKNPELSVQARSLIMEFWECGSLYQYKKAAIINTSNGRIPIKGVLAELIVNWKRE
CidA_I(γ/1)      LSDTMLKNPELSVQARSLIMEFWECGSLYQYKKAAIINTSNGRIPIKGVLAELIVNWKRE
CidA_I(γ/2)      LSDTMLKNPELSVQARSLIMEFWECGSLYQYRKAA-VNTSNYTVPTSGVFAELIVNWRRE
CidA_I(β/2)      LSDTMLKNPELSVQARSLIMEFWECGSLYQYRKAA-VNTSNYTVPTSGVFAELIVNWRRE
CidA_II(α/1)     LSDTMLKNPELSVQARSLIMEIWQCGSFSEYKKAAIINTSNGRIPIKGVLAELIVNWKRE
CidA_II(α/2)     LSDVMLKNPESNVQARSLIMEFWECGSLYQYRKAA-VNTSNYTVPTSGVFAELIVNWRRE
CidA_II(β/2)     LSDVMLKNPESNVQARSLIMEFWECGSLYQYRKAA-VNTSNYTVPTSGVFAELIVNWRRE
CidA_III(α/1)    LSDTMLKNPELSVQARSLIMEFWECGSLYQYRKAAIINTSNGRIPIKGVLAELIVNWKRE
CidA_III(β/2)    LSDTMLKNPELSVQARSLIMEFWECGSLYQYRKAAIINTSNGRIPIKGVLAELIVNWKRE
CidA_III(β/1)    LSDTMLKNPELSVQARSLIMEFWECGSLYQYRKAAIINTSNGRIPIKGVLAELIVNWKRE
CidA_III(β/3)    LSDTMLKNPELSVQARSLIMEFWECGSLYQYRKAA-VNTSNYTVPTSGVFAELIVNWRRE
CidA_IV(α/1)     LSDVMLKNPESNVQARSLIMEFWECGSLYQYRKAAIINTSNGRIPIKGVLAELIVNWKRE
CidA_IV(α/2)     LSDVMLKNPESNVQARSLIMEFWECGSLYQYRKAA-VNTSNYTVPTSGVFAELIVNWRRE
CidA_IV(γ/1)     LSDVMLKNPESNVQARSLIMEFWECGSLYQYRKAAIINTSNGRIPIKGVLAELIVNWKRE
CidA_IV(γ/2)     LSDVMLKNPESNVQARSLIMEFWECGSLYQYRKAA-VNTSNYTVPTSGVFAELIVNWRRE
CidA_IV(δ/1)     LSDVMLKNPESNVQARSLIMEFWECGSLYQYRKAAIINTSNGRIPIKGVLAELIVNWKRE
CidA_IV(δ/2)     LSDVMLKNPESNVQARSLIMEFWECGSLYQYRKAA-VNTSNYTVPTSGVFAELIVNWRRE
```

```
CidA_I(α/1)     DIYKPDEEKEIEKKEILDMMLFAKNSFPERFEFFKEKIIKNLRLCGREGKRVNVDYGLFA
CidA_I(γ/1)     DIYKPDEEKEIEKKEILDMMLFAKNSFPERFEFFKEKIIKNLRLCGREGKRVNVDYGLFA
CidA_I(γ/2)     DIYKTDEEKEIEKKEILDMMSFAKDCFPEKFELFKKLIIRDLRLCGREGKRVNVDYGLFA
CidA_I(β/2)     DIYKTDEEKEIEKKEILDMMSFAKDCFPEKFELFKKLIIRDLRLCGREGKRVNVDYGLFA
CidA_II(α/1)    DIYKPDEEKEIEKKEILDMMLFAKNSFPERFEFFKEKIIKNLRLCGREGEKVNVDYGLFA
CidA_II(α/2)    DIYKTDEEKEIEKKEILDMMSFAKDCFPEKFELFKKLIIRDLRLCGREGKRVNVDYGLFA
CidA_II(β/2)    DIYKTDEEKEIEKKEILDMMSFAKDCFPEKFELFKKLIIRDLRLCGREGKRVNVDYGLFA
CidA_III(α/1)   DIYKPDEEKEIEKKEILDMMLFAKNSFPERFEFFKEKIIKNLRLCGREGKRVNVDYGLFA
CidA_III(β/2)   DIYKPDEEKEIEKKEILDMMLFAKNSFPERFEFFKEKIIKNLRLCGREGEKVNVDYGLFA
CidA_III(β/1)   DIYKPDEEKEIEKKEILDMMLFAKNSFPERFEFFKEKIIKNLRLCGREGKRVNVDYGLFA
CidA_III(β/3)   DIYKTDEEKEIEKKEILDMMSFAKDCFPEKFELFKKLIIRDLRLCGREGKRVNVDYGLFA
CidA_IV(α/1)    DIYKPDEEKEIEKKEILDMMLFAKNSFPERFEFFKEKIIKNLRLCGREGEKVNVDYGLFA
CidA_IV(α/2)    DIYKTDEEKEIEKKEILDMMSFAKDCFPEKFELFKKLIIRDLRLCGREGEKVNVDYGLFA
CidA_IV(γ/1)    DIYKPDEEKEIEKKEILDMMLFAKNSFPERFEFFKEKIIKNLRLCGREGEKVNVDYGLFA
CidA_IV(γ/2)    DIYKTDEEKEIEKKEILDMMSFAKDCFPEKFELFKKLIIRDLRLCGREGEKVNVDYGLFA
CidA_IV(δ/1)    DIYKPDEEKEIEKKEILDMMLFAKNSFPERFEFFKEKIIKNLRLCGREGEKVNVDYGLFA
CidA_IV(δ/2)    DIYKTDEEKEIEKKEILDMMSFAKDCFPEKFELFKKLIIRDLRLCGREGEKVNVDYGLFA

CidA_I(α/1)     EELFSELEKTILPPGPVGDGPCSNLRSRSKAHGSKKTTLPVDDSPQSELGTPSVSGVSSY
CidA_I(γ/1)     EELFSELEKTILPPGPVGDGPCSNLRSRSKAHGSKKTTLPVDDSPQSELGTPSVSGVSSY
CidA_I(γ/2)     EELFSELEKTILPPGPVGDGPCSNLRSRSKAHGSKKTTLPVDDSPQSELGTPSVSGVSSY
CidA_I(β/2)     EELFSELEKTILPPGPVGDGPCSNLRSRSKAHGSKKTTLPVDDSPQSELGTPSVSGVSSY
CidA_II(α/1)    EELFSELEKTILPPGPVGDGPCSNLRSRSKAHGSKKTTLPVDDSPQSELGTPSVSGVSSY
CidA_II(α/2)    EELFSELEKTILPPGPVGDGPCSNLRSRSKAHGSKKTTLPVDDSPQSELGTPSVSGVSSY
CidA_II(β/2)    EELFSELEKTILPPGPVGDGPCSNLRSRSKAHGSKKTTLPVDDSPQSELGTPSVSGVSSY
CidA_III(α/1)   EELFSELEKTILPPGPVGDGPCSNLRSRSKAHGSKKTTLPVDDSPQSELGTPSVSGVSSY
CidA_III(β/2)   EELFSELEKTILPPGPVGDGPCSNLRSRSKAHGSKKTTLPVDDSPQSELGTPSVSGVSSY
CidA_III(β/1)   EELFSELEKTILPPGPVGDGPCSNLRSRSKAHGSKKTTLPVDDSPQSELGTPSVSGVSSY
CidA_III(β/3)   EELFSELEKTILPPGPVGDGPCSNLRSRSKAHGSKKTTLPVDDSPQAELGTPSVSGVSSY
CidA_IV(α/1)    EELFSELEKTTLP--PVGDGPCSNLRSRSKAHGSKKTTFPVDDSPQSELGTPSVSGVSSY
CidA_IV(α/2)    EELFSELEKTTLP--PVGDGPCSNLRSRSKAHGSKKTTFPVDDSPQSELGTPSVSGVSSY
CidA_IV(γ/1)    EELFSELEKTTLP--PVGDGPCSNLRSRSKAHGSKKTTFPVDDSPQSELGTPSVSGVSSY
CidA_IV(γ/2)    EELFSELEKTTLP--PVGDGPCSNLRSRSKAHGSKKTTFPVDDSPQSELGTPSVSGVSSY
CidA_IV(δ/1)    EELFSELEKTTLP--PVGDGPCSNLRSRSKAHGSKKTTFPVDDSPQSELGTPSVSGVSSY
CidA_IV(δ/2)    EELFSELEKTTLP--PVGDGPCSNLRSRSKAHGSKKTTFPVDDSPQSELGTPSVSGVSSY

CidA_I(α/1)     KKKSVFTLSGNK
CidA_I(γ/1)     KKKSVFTLSGNK
CidA_I(γ/2)     KKKSVFTLSGNK
CidA_I(β/2)     KKKSVFTLSGNK
CidA_II(α/1)    KKKSVFTLSGNK
CidA_II(α/2)    KKKSVFTLSGNK
CidA_II(β/2)    KKKSVFTLSGNK
CidA_III(α/1)   KKKSVFTLSGNK
CidA_III(β/2)   KKKSVFTLSGNK
CidA_III(β/1)   KKKSVFTLSGNK
CidA_III(β/3)   KKKSVFTLSGNK
CidA_IV(α/1)    KKKSVFTLSGNK
CidA_IV(α/2)    KKKSVFTLSGNK
CidA_IV(γ/1)    KKKSVFTLSGNK
CidA_IV(γ/2)    KKKSVFTLSGNK
CidA_IV(δ/1)    KKKSVFTLSGNK
CidA_IV(δ/2)    KKKSVFTLSGNK
```

Supplementary Figure 2 | Complete sequences of the CidA variants from the different *w*Pip groups (I to IV).

Polymorphic regions represented by colored blocks in Figure 3 are reported on the alignment.

```
CidB_I(a/1)      MSNGDGLIRSLVDGDLEGFRQGFESFLDQCPSFLYHVSAGRFLPVFFFSMFSTAHDANIL
CidB_I(a/2)      MSNGDGLIRSLVDGDLEGFRQGFESFLDQCPSFLYHVSAGRFLPVFFFSMFSTAHDANIL
CidB_I(b/1)      MSNGDGLIRSLVDGDLEGFRQGFESFLDQCPSFLYHVSAGRFLPVFFFSMFSTAHDANIL
CidB_I(b/2)      MSNGDGLIRSLVDGDLEGFRQGFESFLDQCPSFLYHVSAGRFLPVFFFSMFSTAHDANIL
CidB_II(a/2)     MSNGDGLIRSLVDGDLEGFRQGFESFLDQCPSFLYHVSAGRFLPVFFFSMFSTAHDANIL
CidB_II(a/1)     MSNGDGLIRSLVDGDLEGFRQGFESFLDQCPSFLYHVSAGRFLPVFFFSMFSTAHDANIL
CidB_III(c/1)    MSNGDGLIRSLVDGDLEGFRQGFESFLDQCPSFLYHVSAGRFLPVFFFSMFSTAHDANIL
CidB_III(a/1)    MSNGDGLIRSLVDGDLEGFRQGFESFLDQCPSFLYHVSAGRFLPVFFFSMFSTAHDANIL
CidB_III(b/1)    MSNGDGLIRSLVDGDLEGFRQGFESFLDQCPSFLYHVSAGRFLPVFFFSMFSTAHDANIL
CidB_IV(a/1)     MSNGDGLIRSLVDGDLEGFRQGFESFLDQCPSFLYHVSAGRFLPVFFFSMFSTAHDANIL
CidB_IV(a/2)     MSNGDGLIRSLVDGDLEGFRQGFESFLDQCPSFLYHVSAGRFLPVFFFSMFSTAHDANIL
CidB_IV(b/1)     MSNGDGLIRSLVDGDLEGFRQGFESFLDQCPSFLYHVSAGRFLPVFFFSMFSTAHDANIL
CidB_IV(b/2)     MSNGDGLIRSLVDGDLEGFRQGFESFLDQCPSFLYHVSAGRFLPVFFFSMFSTAHDANIL


CidB_I(a/1)      NANERVYFRFDNHGVNPRNGENRNTANLKVAVYRDGQQVVRCYSISDRPNSDGLRFSERE
CidB_I(a/2)      NANERVYFRFDNHGVNPRNGENRNTANLKVAVYRDGQQVVRCYSISDRPNSDGLRFSERE
CidB_I(b/1)      NANERVYFRFDNHGVNPRNGENRNTANLKVAVYRDGQQVVRCYSISDRPNSDGLRFSTRE
CidB_I(b/2)      NANERVYFRFDNHGVNPRNGENRNTANLKVAVYRDGQQVVRCYSISDRPNSDGLRFSTRE
CidB_II(a/2)     NANERVYFRFDNHGVNPRNGENRNTANLKVAVYRDGQQVVRCYSISDRPNSDGLRFSERE
CidB_II(a/1)     NANERVYFRFDNHGVNPRNGENRNTANLKVAVYRDGQQVVRCYSISDRPNSDGLRFSERE
CidB_III(c/1)    NANERVYFRFDNHGVNPRNGENRNTANLKVAVYRDGQQVVRCYSISDRPNSDGLRFSERE
CidB_III(a/1)    NANERVYFRFDNHGVNPRNGENRNTANLKVAVYRDGQQVVRCYSISDRPNSDGLRFSERE
CidB_III(b/1)    NANERVYFRFDNHGVNPRNGENRNTANLKVAVYRDGQQVVRCYSISDRPNSDGLRFSERE
CidB_IV(a/1)     NANERVYFRFDNHGVNPRNGENRNTANLKVAVYRDGQQVVRCYSISDRPNSDGLRFSERE
CidB_IV(a/2)     NANERVYFRFDNHGVNPRNGENRNTANLKVAVYRDGQQVVRCYSISDRPNSDGLRFSERE
CidB_IV(b/1)     NANERVYFRFDNHGVNPRNGENRNTANLKVAVYRDGQQVVRCYSISDRPNSDGLRFSERE
CidB_IV(b/2)     NANERVYFRFDNHGVNPRNGENRNTANLKVAVYRDGQQVVRCYSISDRPNSDGLRFSERE


CidB_I(a/1)      RDFLVQEIIRQNQGLMEEDLNFEQYKVCMHGKGKSQGEAIATVFEVIREKDFRGRDKFAK
CidB_I(a/2)      RDFLVQEIIRQNQGLMEEDLNFEQYKVCMHGKGKSQGEAIATVFEVIREKDFRGRDKFAK
CidB_I(b/1)      RNALVQEIRRQNPNLREEDLNFEQYKVCMHGKGKSQGEAIATVFEVIREKDRQGRDKFAK
CidB_I(b/2)      RNALVQEIRRQNPNLREEDLNFEQYKVCMHGKGKSQGEAIATVFEVIREKDRQGRDKFAK
CidB_II(a/2)     RDFLVQEIIRQNQGLMEEDLNFEQYKVCMHGKGKSQGEAIATVFEVIREKDRQGRDKFAK
CidB_II(a/1)     RDFLVQEIIRQNQGLMEEDLNFEQYKVCMHGKGKSQGEAIATVFEVIREKDRQGRDKFAK
CidB_III(c/1)    RDFLVQEIIRQNQGLMEEDLNFEQYKVCMHGKGKSQGEAIATVFEVIREKDFRGRDKFAK
CidB_III(a/1)    RDFLVQEIIRQNQGLMEEDLNFEQYKVCMHGKGKSQGEAIATVFEVIREKDFRGRDKFAK
CidB_III(b/1)    RDFLVQEIIRQNQGLMEEDLNFEQYKVCMHGKGKSQGEAIATVFEVIREKDFRGRDKFAK
CidB_IV(a/1)     RDFLVQEIIRQNQGLMEEDLNFEQYKVCMHGKGKSQGEAIATVFEVIREKDFRGRDKFAK
CidB_IV(a/2)     RDFLVQEIIRQNQGLMEEDLNFEQYKVCMHGKGKSQGEAIATVFEVIREKDFRGRDKFAK
CidB_IV(b/1)     RDFLVQEIIRQNQGLMEEDLNFEQYKVCMHGKGKSQGEAIATVFEVIREKDFRGRDKFAK
CidB_IV(b/2)     RDFLVQEIIRQNQGLMEEDLNFEQYKVCMHGKGKSQGEAIATVFEVIREKDFRGRDKFAK


CidB_I(a/1)      YSASEVHFLRQLFRNHRLTIKEIEGRQLNQNQLRQLGRSVNFTRVEPGQQRIDNFMEMLA
CidB_I(a/2)      YSASEVHFLRQLFRNHRLTIKEIEGRQLNQNQLRQLGRSVNFTRVEPGQQRIDNFMEMLA
CidB_I(b/1)      YSASEINLIRRLLGDHRLTIKEIEGRQLNQNQLRQLGRLVNFAQVAQGQQGIDNFMEMLA
CidB_I(b/2)      YSASEINLIRRLLGDHRLTIKEIEGRQLNQNQLRQLGRLVNFAQVAQGQQGIDNFMEMLA
CidB_II(a/2)     YSASEINLIRRLLGDHRLTIKEIEGRQLNQNQRRQLGRLVNFAQVAQGQQGIDNFMEMLA
CidB_II(a/1)     YSASEINLIRRLLGDHRLTIKEIEGRQLNQNQRRQLGRLVNFAQVAQGQQGIDNFMEMLA
CidB_III(c/1)    YSASEVHFLRQLFRNHRLTIKEIEGRQLNQNQRRQLGRLVNFAQVAQGQQGIDNFMEMLA
CidB_III(a/1)    YSASEVHFLRQLFRNHRLTIKEIGGRQLNQNQLRQLGRSVNFTRVEPGQQRIDNFMEMLA
CidB_III(b/1)    YSASEVHFLRQLFRNHRLTIKEIEGRQLNQNQRRQLGRLVNFAQVAQGQQGIDNFMEMLA
CidB_IV(a/1)     YSASEINLIRRLLGDHRLTIKEIEGRQLNQNQRRQLGRLVNFAQVAQGQQGIDNFMEMLA
CidB_IV(a/2)     YSASEINLIRRLLGDHRLTIKEIEGRQLNQNQRRQLGRLVNFAQVAQGQQGIDNFMEMLA
CidB_IV(b/1)     YSASEINLIRRLLGDHRLTIKEIEGRQLNQNQRRQLGRLVNFAQVAQGQQGIDNFMEMLA
CidB_IV(b/2)     YSASEINLIRRLLGDHRLTIKEIEGRQLNQNQRRQLGRLVNFAQVAQGQQGIDNFMEMLA
```

```
CidB_I(a/1)      SNQRQDVRDSLRGDILEYVTDTYNNYRAQIENNIEGRSQKFESHGFLLGFLANFSHRYTI
CidB_I(a/2)      SNQRQDVRDSLRGDILEYVTDTYNNYRAQIENNIEGRSQKFESHGFLLGFLANFSHRYTI
CidB_I(b/1)      SDRRQDVRDRIRREILPYITDIYNNYRQVLENNIENRNQRFEGHGFLLGFLANFSHRYTI
CidB_I(b/2)      SDRRQDVRDRIRREILPYITDIYNNYRQVLENNIENRNQRFEGHGFLLGFLANFSHRYTI
CidB_II(a/2)     SDRRQDVRDRIRREILPYITDIYNNYRQVLENNIENRNQRFEGHGFLLGFLANFSHRYTI
CidB_II(a/1)     SDRRQDVRDRIRREILPYITDIYNNYRQVLENNIENRNQRFEGHGFLLGFLANFSHRYTI
CidB_III(c/1)    SDRRQDVRDRIRREILPYITDIYNNYRQVLENNIENRNQRFEGHGFLLGFLANFSHLCTI
CidB_III(a/1)    SNQRQDVRDSLRGDILEYVTDTYNNYRAQIENNIEGRSQKFESHGFLLGFLANFSHRYTI
CidB_III(b/1)    SDRRQDVRDRIRREILPYITDIYNNYRQVLENNIENRNQRFEGHGFLLGFLANFSHRYTI
CidB_IV(a/1)     SDRRQDVRDRIRREILPYITDIYNNYRQVLENNIENRNQRFEGHGFLLGFLANFSHLCTI
CidB_IV(a/2)     SDRRQDVRDRIRREILPYITDIYNNYRQVLENNIENRNQKFESHGFLLGFLANFSHLCTI
CidB_IV(b/1)     SNQRQDVRDSLRGDILEYVTDTYNNYRAQIENNIEGRSQKFESHGFLLGFLANFSHLCTI
CidB_IV(b/2)     SNQRQDVRDSLRGDILEYVTDTYNNYRAQIENNIEGRSQKFESHGFLLGFLANFSHLCTI
```

```
CidB_I(a/1)      GVDLDLSPRNSHVAFLVRHQVERENIPIVINLATRA-PPYIALNRARSHAERLHVFSFIP
CidB_I(a/2)      GVDLDLSPRNSHVAFLVRHQVERENIPIVINLATRA-PPYIALNRARSHAERLHVFSFIP
CidB_I(b/1)      GVDLDLSPRNSHVAFLVRHQVERENIPIVINLATRA-PPYIALNRARSHAERLHVFSFIP
CidB_I(b/2)      GVDLDLSPRNSHVAFLVRHQVERENIPIVINLATRA-PPYIALNRARSHAERLHVFSFIP
CidB_II(a/2)     GVDLDLSPRNSHVAFLVRHQVERENIPIVINLATRA-PPYIALNRARSHAERLHVFSFIP
CidB_II(a/1)     GVDLDLSPRNSHVAFLVRHQVERENIPIVINLATRA-PPYIALNRARSHAERLHVFSFIP
CidB_III(c/1)    DIDLDLSPRNSHVAFLVRHQVERENIPIVINLATRA-PPYIALNRARSHAERLHVFSFIP
CidB_III(a/1)    GVDLDLSPRNSHVAFLVRHQVERENIPIVINLATRA-PPYIALNRARSHAERLHVFSFIP
CidB_III(b/1)    GVDLDLSPRNSHVAFLVRHQVERENIPIVINLATRA-PPYIALNRARSHAERLHVFSFIP
CidB_IV(a/1)     DIDLDLSPRNSHVAFLVRHQAERENIPIVINLAIFDTPSDVALNCAIGYAGRLHTSSFIP
CidB_IV(a/2)     DIDLDLSPRNSHVAFLVRHQAERENIPIVINLAIFDTPSDVALNCAIGYAGRLHTSSFIP
CidB_IV(b/1)     DIDLDLSPRNSHVAFLVRHQAERENIPIVINLAIFDTPSDVALNCAIGYAGRLHTSSFIP
CidB_IV(b/2)     DIDLDLSPRNSHVAFLVRHQAERENIPIVINLAIFDTPSDVALNCAIGYAGRLHTSSFIP
```

```
CidB_I(a/1)      IHTESRNTVCVGLNFNLNLDPFSVDTVGLQQDRFPLVQRLFECLENEGIRENIRDFLLHH
CidB_I(a/2)      IHTESRNTVCVGLNFNLNLDPFSVDTVGLQQDRFPLVQRLFECLENEGIRENIRDFLLHH
CidB_I(b/1)      IHTESRNTVCVGLNFNLNLDPFSVDTVGLQQDRFPLVQRLFECLENEGIRENIRDFLLHH
CidB_I(b/2)      IHTESRNTVCVGLNFNLNLDPFSVDTVGLQQDRFPLVQRLFECLENEGIRENIRDFLLHH
CidB_II(a/2)     IHTESRNTVCVGLNFNLNLDPFSVDTVGLQQDRFPLVQRLFECLENEGIRENIRDFLLHH
CidB_II(a/1)     IHTESRNTVCVGLNFNLNLDPFSVDTVGLQQDRFPLVQRLFECLENEGIRENIRDFLLHH
CidB_III(c/1)    IHTESRNTVCVGLNFNLNLDPFSVDTVGLQQDRFPLVQRLFECLENEGIRENIRDFLLHH
CidB_III(a/1)    IHTESRNTVCVGLNFNLNLDPFSVDTVGLQQDRFPLVQRLFECLENEGIRENIRDFLLHH
CidB_III(b/1)    IHTESRNTVCVGLNFNLNLDPFSVDTVGLQQDRFPLVQRLFECLENEGIRENIRDFLLHH
CidB_IV(a/1)     IHTTSEHTVCVGLNFNSRLAPFRVGIAELQQNRFPLVQRLFECLENEGIRENIRDFLLHH
CidB_IV(a/2)     IHTTSEHTVCVGLNFNSRLAPFRVGIAELQQNRFPLVQRLFECLENEGIRENIRDFLLHH
CidB_IV(b/1)     IHTTSEHTVCVGLNFNSRLAPFRVGIAELQQNRFPLVQRLFECLENEGIRENIRDFLLHH
CidB_IV(b/2)     IHTTSEHTVCVGLNFNSRLAPFRVGIAELQQNRFPLVQRLFECLENEGIRENIRDFLLHH
```

```
CidB_I(a/1)      LPAEIPRNAENYDRIFDCITGFAFGNSAFDREYLELEEGEDRVRVRKYIFRYGDGDLRRH
CidB_I(a/2)      LPAEIPRNAENYDRIFDCITGFAFGNSAFDRHPLELEE-EDEAPITKYIFRHGDEGLR-C
CidB_I(b/1)      LPAEIPRNAENYDRIFDCITGFAFGNSAFDREYLELEEGEDRVRVRKYIFRYGDGDLRRH
CidB_I(b/2)      LPAEIPRNAENYDRIFDCITGFAFGNSAFDRHPLELEE-EDEAPITKYIFRHGDEGLR-C
CidB_II(a/2)     LPAEIPRNAENYDRIFDCITGFAFGNSAFDRHPLELEE-EDEAPITKYIFRHGDEGLR-C
CidB_II(a/1)     LPAEIPRNAENYDRIFDCITGFAFGNSAFDREYLELEEGEDRVRVRKYIFRYGDGDLRRH
CidB_III(c/1)    LPAEIPRNAENYDRIFDCITGFAFGNSAFDREYLELEEGEDRVRVRKYIFRYGDGDLRRH
CidB_III(a/1)    LPAEIPRNAENYDRIFDCITGFAFGNSAFDREYLELEEGEDRVRVRKYIFRYGDGDLRRH
CidB_III(b/1)    LPAEIPRNAENYDRIFDCITGFAFGNSAFDREYLELEEGEDRVRVRKYIFRYGDGDLRRH
CidB_IV(a/1)     LPAEIPRNAENYDRIFDCITGFAFGNSAFDREYLELEEGEDRVRVRKYIFRYGDGDLRRH
CidB_IV(a/2)     LPAEIPRNAENYDRIFDCITGFAFGNSAFDREYLELEE-EDEAPITKYIFRHGDEGLR-C
CidB_IV(b/1)     LPAEIPRNAENYDRIFDCITGFAFGNSAFDREYLELEEGEDRVRVRKYIFRYGDGDLRRH
CidB_IV(b/2)     LPAEIPRNAENYDRIFDCITGFAFGNSAFDREYLELEE-EDEAPITKYIFRHGDEGLR-C
```

```
CidB_I(a/1)        TLTMVFHAEGSDIVILHIRAHDAQQQGAINLQTLNVNGNDVHVWEVSCTLNNQLELDIDL
CidB_I(a/2)        -LTMVFHAEGSDIVILHIRAHDAQQQGAINLQTLNVNGNDVHVWEVSCTLNNQLELDIDL
CidB_I(b/1)        TLTMVFHAEGSDIVILHIRAHDAQQQGAINLQTLNVNGNDVHVWEVSCTLNNQLELDIDL
CidB_I(b/2)        -LTMVFHAEGSDIVILHIRAHDAQQQGAINLQTLNVNGNDVHVWEVSCTLNNQLELDIDL
CidB_II(a/2)       -LTMVFHAEGSDIVILHIRAHDAQQQGAINLQTLNVNGNDVHVWEVSCTLNNQLELDIDL
CidB_II(a/1)       TLTMVFHAEGSDIVILHIRAHDAQQQGAINLQTLNVNGNDVHVWEVSCTLNNQLELDIDL
CidB_III(c/1)      TLTMVFHAEGSDIVILHIRAHDAQQQGAINLQTLNVNGNDVHVWEVSCTLNNQLELDIDL
CidB_III(a/1)      TLTMVFHAEGSDIVILHIRAHDAQQQGAINLQTLNVNGNDVHVWEVSCTLNNQLELDIDL
CidB_III(b/1)      TLTMVFHAEGSDIVILHIRAHDAQQQGAINLQTLNVNGNDVHVWEVSCTLNNQLELDIDL
CidB_IV(a/1)       TLTMVFHAEGSDIVILHIRAHDAQQQGAINLQTLNVNGNDVHVWEVSCTLNNQLELDIDL
CidB_IV(a/2)       -LTMVFHAEGSDIVILHIRAHDAQQQGAINLQTLNVNGNDVHVWEVSCTLNNQLELDIDL
CidB_IV(b/1)       TLTMVFHAEGSDIVILHIRAHDAQQQGAINLQTLNVNGNDVHVWEVSCTLNNQLELDIDL
CidB_IV(b/2)       -LTMVFHAEGSDIVILHIRAHDAQQQGAINLQTLNVNGNDVHVWEVSCTLNNQLELDIDL


CidB_I(a/1)        PNDLGLYHDYQNNNANNFLAGDLVQVPNTENVHNTLNQVVNDGWKNIAQHRGLFQEISGA
CidB_I(a/2)        PNDLGLYHDYQNNNANNFLAGDLVQVPNTENVHNTLNQVVNDGWKNIAQHRGLFQEISGA
CidB_I(b/1)        PNDLGLYHDYQNNNANNFLAGDLVQVPNTENVHNTLNQVVNDGWKNIAQHRGLFQEISGA
CidB_I(b/2)        PNDLGLYHDYQNNNANNFLAGDLVQVPNTENVHNTLNQVVNDGWKNIAQHRGLFQEISGA
CidB_II(a/2)       PNDLGLYHDYQNNNANNFLAGDLVQVPNTENVHNTLNQVVNDGWKNIAQHRGLFQEISGA
CidB_II(a/1)       PNDLGLYHDYQNNNANNFLAGDLVQVPNTENVHNTLNQVVNDGWKNIAQHRGLFQEISGA
CidB_III(c/1)      PNDLGLYHDYQNNNANNFLAGDLVQVPNTENVHNTLNQVVNDGWKNIAQHRGLFQEISGA
CidB_III(a/1)      PNDLGLYHDYQNNNANNFLAGDLVQVPNTENVHNTLNQVVNDGWKNIAQHRGLFQEISGA
CidB_III(b/1)      PNDLGLYHDYQNNNANNFLAGDLVQVPNTENVHNTLNQVVNDGWKNIAQHRGLFQEISGA
CidB_IV(a/1)       PNDLGLYHDYQNNNANNFLAGDLVQVPNTENVHNTLNQVVNDGWKNIAQHRGLFQEISGA
CidB_IV(a/2)       PNDLGLYHDYQNNNANNFLAGDLVQVPNTENVHNTLNQVVNDGWKNIAQHRGLFQEISGA
CidB_IV(b/1)       PNDLGLYHDYQNNNANNFLAGDLVQVPNTENVHNTLNQVVNDGWKNIAQHRGLFQEISGA
CidB_IV(b/2)       PNDLGLYHDYQNNNANNFLAGDLVQVPNTENVHNTLNQVVNDGWKNIAQHRGLFQEISGA


CidB_I(a/1)        LMPLVDTINVNSEDKFRSILHGTFYASDNPYKVLAMYKVGQTYSLKRGQEEEGERVILTR
CidB_I(a/2)        LMPLVDTINVNSEDKFRSILHGTFYASDNPYKVLAMYKVGQTYSLKRGQEEEGERVILTR
CidB_I(b/1)        LMPLVDTINVNSEDKFRSILHGTFYASDNPYKVLAMYKVGQTYSLKRGQEEEGERVILTR
CidB_I(b/2)        LMPLVDTINVNSEDKFRSILHGTFYASDNPYKVLAMYKVGQTYSLKRGQEEEGERVILTR
CidB_II(a/2)       LMPLVDTINVNSEDKFRSILHGTFYASDNPYKVLAMYKVGQTYSLKRGQEEEGERVILTR
CidB_II(a/1)       LMPLVDTINVNSEDKFRSILHGTFYASDNPYKVLAMYKVGQTYSLKRGQEEEGERVILTR
CidB_III(c/1)      LMPLVDTINVNSEDKFRSILHGTFYASDNPYKVLAMYKVGQTYSLKRGQEEEGERVILTR
CidB_III(a/1)      LMPLVDTINVNSEDKFRSILHGTFYASDNPYKVLAMYKVGQTYSLKRGQEEEGERVILTR
CidB_III(b/1)      LMPLVDTINVNSEDKFRSILHGTFYASDNPYKVLAMYKVGQTYSLKRGQEEEGERVILTR
CidB_IV(a/1)       LMPLVDTINVNSEDKFRSILHGTFYASDNPYKVLAMYKVGQTYSLKRGQEEEGERVILTR
CidB_IV(a/2)       LMPLVDTINVNSEDKFRSILHGTFYASDNPYKVLAMYKVGQTYSLKRGQEEEGERVILTR
CidB_IV(b/1)       LMPLVDTINVNSEDKFRSILHGTFYASDNPYKVLAMYKVGQTYSLKRGQEEEGERVILTR
CidB_IV(b/2)       LMPLVDTINVNSEDKFRSILHGTFYASDNPYKVLAMYKVGQTYSLKRGQEEEGERVILTR


CidB_I(a/1)        ITEQRLDLLLLRQPRENDLDTHPIGYVLRLANNAEEVGQQQNDARQEIGRLKKQHRGFIP
CidB_I(a/2)        ITEQRLDLLLLRQPRENDLDTHPIGYVLRLANNAEEVGQQQNDARQEIGRLKKQHRGFIP
CidB_I(b/1)        ITEQRLDLLLLRQPRENDLDTHPIGYVLRLANNAEEVGQQQNDARQEIGRLKKQHRGFIP
CidB_I(b/2)        ITEQRLDLLLLRQPRENDLDTHPIGYVLRLANNAEEVGQQQNDARQEIGRLKKQHRGFIP
CidB_II(a/2)       ITEQRLDLLLLRQPRENDLDTHPIGYVLRLANNAEEVGQQQNDARQEIGRLKKQHRGFIP
CidB_II(a/1)       ITEQRLDLLLLRQPRENDLDTHPIGYVLRLANNAEEVGQQQNDARQEIGRLKKQHRGFIP
CidB_III(c/1)      ITEQRLDLLLLRQPRENDLDTHPIGYVLRLANNAEEVGQQQNDARQEIGRLKKQHRGFIP
CidB_III(a/1)      ITEQRLDLLLLRQPRENDLDTHPIGYVLRLANNAEEVGQQQNDARQEIGRLKKQHRGFIP
CidB_III(b/1)      ITEQRLDLLLLRQPRENDLDTHPIGYVLRLANNAEEVGQQQNDARQEIGRLKKQHRGFIP
CidB_IV(a/1)       ITEQRLDLLLLRQPRENDLDTHPIGYVLRLANNAEEVGQQQNDARQEIGRLKKQHRGFIP
CidB_IV(a/2)       ITEQRLDLLLLRQPRENDLDTHPIGYVLRLANNAEEVGQQQNDARQEIGRLKKQHRGFIP
CidB_IV(b/1)       ITEQRLDLLLLRQPRENDLDTHPIGYVLRLANNAEEVGQQQNDARQEIGRLKKQHRGFIP
CidB_IV(b/2)       ITEQRLDLLLLRQPRENDLDTHPIGYVLRLANNAEEVGQQQNDARQEIGRLKKQHRGFIP
```

```
CidB_I(a/1)        ITSGNEVVLFPIVFNRDAHEAGNLILFPEGIGREEHVHRLDRHVRSSRPGGLVGPESVID
CidB_I(a/2)        ITSGNEVVLFPIVFNRDAHEAGNLILFPEGIGREEHVHRLDRHVRSSRPGGLVGPESVID
CidB_I(b/1)        ITSGNEVVLFPIVFNRDAHEAGNLILFPEGIGREEHVHRLDRHVRSSRPGGLVGPESVID
CidB_I(b/2)        ITSGNEVVLFPIVFNRDAHEAGNLILFPEGIGREEHVHRLDRHVRSSRPGGLVGPESVID
CidB_II(a/2)       ITSGNEVVLFPIVFNRDAHEAGNLILFPEGIGREEHVHRLDRHVRSSRPGGLVGPESVID
CidB_II(a/1)       ITSGNEVVLFPIVFNRDAHEAGNLILFPEGIGREEHVHRLDRHVRSSRPGGLVGPESVID
CidB_III(c/1)      ITSGNEVVLFPIVFNRDAHEAGNLILFPEGIGREEHVHRLDRHVRSSRPGGLVGPESVID
CidB_III(a/1)      ITSGNEVVLFPIVFNRDAHEAGNLILFPEGIGREEHVHRLDRHVRSSRPGGLVGPESVID
CidB_III(b/1)      ITSGNEVVLFPIVFNRDAHEAGNLILFPEGIGREEHVHRLDRHVRSSRPGGLVGPESVID
CidB_IV(a/1)       ITSGNEVVLFPIVFNRDAHEAGNLILFPEGIGREEHVHRLDRHVRSSRPGGLVGPESVID
CidB_IV(a/2)       ITSGNEVVLFPIVFNRDAHEAGNLILFPEGIGREEHVHRLDRHVRSSRPGGLVGPESVID
CidB_IV(b/1)       ITSGNEVVLFPIVFNRDAHEAGNLILFPEGIGREEHVHRLDRHVRSSRPGGLVGPESVID
CidB_IV(b/2)       ITSGNEVVLFPIVFNRDAHEAGNLILFPEGIGREEHVHRLDRHVRSSRPGGLVGPESVID


CidB_I(a/1)        ENPPEGLLSDQTRENFRRFYEEKAPGQNSIFLLDIGDNLHVPFSYLQGTRAQVIETLKSR
CidB_I(a/2)        ENPPEGLLSDQTRENFRRFYEEKAPGQNSIFLLDIGDNLHVPFSYLQGTRAQVIETLKSR
CidB_I(b/1)        ENPPEGLLSDQTRENFRRFYEEKAPGQNSIFLLDIGDNLHVPFSYLQGTRAQVIETLKSR
CidB_I(b/2)        ENPPEGLLSDQTRENFRRFYEEKAPGQNSIFLLDIGDNLHVPFSYLQGTRAQVIETLKSR
CidB_II(a/2)       ENPPEGLLSDQTRENFRRFYEEKAPGQNSIFLLDIGDNLHVPFSYLQGTRAQVIETLKSR
CidB_II(a/1)       ENPPEGLLSDQTRENFRRFYEEKAPGQNSIFLLDIGDNLHVPFSYLQGTRAQVIETLKSR
CidB_III(c/1)      ENPPEGLLSDQTRENFRRFYEEKAPGQNSIFLLDIGDNLHVPFSYLQGTRAQVIETLKSR
CidB_III(a/1)      ENPPEGLLSDQTRENFRRFYEEKAPGQNSIFLLDIGDNLHVPFSYLQGTRAQVIETLKSR
CidB_III(b/1)      ENPPEGLLSDQTRENFRRFYEEKAPGQNSIFLLDIGDNLHVPFSYLQGTRAQVIETLKSR
CidB_IV(a/1)       ENPPEGLLSDQTRENFRRFYEEKAPGQNSIFLLDIGDNLHVPFSYLQGTRAQVIETLKSR
CidB_IV(a/2)       ENPPEGLLSDQTRENFRRFYEEKAPGQNSIFLLDIGDNLHVPFSYLQGTRAQVIETLKSR
CidB_IV(b/1)       ENPPEGLLSDQTRENFRRFYEEKAPGQNSIFLLDIGDNLHVPFSYLQGTRAQVIETLKSR
CidB_IV(b/2)       ENPPEGLLSDQTRENFRRFYEEKAPGQNSIFLLDIGDNLHVPFSYLQGTRAQVIETLKSR


CidB_I(a/1)        IRGGGTPTAQGILQQINAILRRNNAREIEDVHDLLALDFATDNQNYRYWLQTHDMFFAAR
CidB_I(a/2)        IRGGGTPTAQGILQQINAILRRNNAREIEDVHDLLALDFATDNQNYRYWLQTHDMFFAAR
CidB_I(b/1)        IRGGGTPTAQGILQQINAILRRNNAREIEDVHDLLALDFATDNQNYRYWLQTHDMFFAAR
CidB_I(b/2)        IRGGGTPTAQGILQQINAILRRNNAREIEDVHDLLALDFATDNQNYRYWLQTHDMFFAAR
CidB_II(a/2)       IRGGGTPTAQGILQQINAILRRNNAREIEDVHDLLALDFATDNQNYRYWLQTHDMFFAAR
CidB_II(a/1)       IRGGGTPTAQGILQQINAILRRNNAREIEDVHDLLALDFATDNQNYRYWLQTHDMFFAAR
CidB_III(c/1)      IRGGGTPTAQGILQQINAILRRNNAREIEDVHDLLALDFATDNQNYRYWLQTHDMFFAAR
CidB_III(a/1)      IRGGGTPTAQGILQQINAILRRNNAREIEDVHDLLALDFATDNQNYRYWLQTHDMFFAAR
CidB_III(b/1)      IRGGGTPTAQGILQQINAILRRNNAREIEDVHDLLALDFATDNQNYRYWLQTHDMFFAAR
CidB_IV(a/1)       IRGGGTPTAQGILQQINAILRRNNAREIEDVHDLLALDFATDNQNYRYWLQTHDMFFAAR
CidB_IV(a/2)       IRGGGTPTAQGILQQINAILRRNNAREIEDVHDLLALDFATDNQNYRYWLQTHDMFFAAR
CidB_IV(b/1)       IRGGGTPTAQGILQQINAILRRNNAREIEDVHDLLALDFATDNQNYRYWLQTHDMFFAAR
CidB_IV(b/2)       IRGGGTPTAQGILQQINAILRRNNAREIEDVHDLLALDFATDNQNYRYWLQTHDMFFAAR


CidB_I(a/1)        QYTFLDNQSHSTNDHYGFEITSVGVDGNQNDPTGRGLLSSHITNFKQKVDSGEKDRLIAI
CidB_I(a/2)        QYTFLDNQSHSTNDHYGFEITSVGVDGNQNDPTGRGLLSSHITNFKQKVDSGEKDRLIAI
CidB_I(b/1)        QYTFLDNQSHSTNDHYGFEITSVGVDGNQNDPTGRGLLSSHITNFKQKVDSGEKDRLIAI
CidB_I(b/2)        QYTFLDNQSHSTNDHYGFEITSVGVDGNQNDPTGRGLLSSHITNFKQKVDSGEKDRLIAI
CidB_II(a/2)       QYTFLDNQSHSTNDHYGFEITSVGVDGNQNDPTGRGLLSSHITNFKQKVDSGEKDRLIAI
CidB_II(a/1)       QYTFLDNQSHSTNDHYGFEITSVGVDGNQNDPTGRGLLSSHITNFKQKVDSGEKDRLIAI
CidB_III(c/1)      QYTFLDNQSHSTNDHYGFEITSVGVDGNQNDPTGRGLLSSHITNFKQKVDSGEKDRLIAI
CidB_III(a/1)      QYTFLDNQSHSTNDHYGFEITSVGVDGNQNDPTGRGLLSSHITNFKQKVDSGEKDRLIAI
CidB_III(b/1)      QYTFLDNQSHSTNDHYGFEITSVGVDGNQNDPTGRGLLSSHITNFKQKVDSGEKDRLIAI
CidB_IV(a/1)       QYTFLDNQSHSTNDHYGFEITSVGVDGNQNDPTGRGLLSSHITNFKQKVDSGEKDRLIAI
CidB_IV(a/2)       QYTFLDNQSHSTNDHYGFEITSVGVDGNQNDPTGRGLLSSHITNFKQKVDSGEKDRLIAI
CidB_IV(b/1)       QYTFLDNQSHSTNDHYGFEITSVGVDGNQNDPTGRGLLSSHITNFKQKVDSGEKDRLIAI
CidB_IV(b/2)       QYTFLDNQSHSTNDHYGFEITSVGVDGNQNDPTGRGLLSSHITNFKQKVDSGEKDRLIAI
```

```
CidB_I(a/1)       INVGNRHWVTLVIVHQNGNYYGYYADSLGPDSGIDNNIRGALRECDINDDNVHNISVHQQ
CidB_I(a/2)       INVGNRHWVTLVIVHQNGNYYGYYADSLGPDSGIDNNIRGALRECDINDDNVHNISVHQQ
CidB_I(b/1)       INVGNRHWVTLVIVHQNGNYYGYYADSLGPDSGIDNNIRGALRECDINDDNVHNISVHQQ
CidB_I(b/2)       INVGNRHWVTLVIVHQNGNYYGYYADSLGPDSGIDNNIRGALRECDINDDNVHNISVHQQ
CidB_II(a/2)      INVGNRHWVTLVIVHQNGNYYGYYADSLGPDSGIDNNIRGALRECDINDDNVHNISVHQQ
CidB_II(a/1)      INVGNRHWVTLVIVHQNGNYYGYYADSLGPDSGIDNNIRGALRECDINDDNVHNISVHQQ
CidB_III(c/1)     INVGNRHWVTLVIVHQNGNYYGYYADSLGPDSGIDNNIRGALRECDINDDNVHNISVHQQ
CidB_III(a/1)     INVGNRHWVTLVIVHQNGNYYGYYADSLGPDSGIDNNIRGALRECDINDDNVHNISVHQQ
CidB_III(b/1)     INVGNRHWVTLVIVHQNGNYYGYYADSLGPDSGIDNNIRGALRECDINDDNVHNISVHQQ
CidB_IV(a/1)      INVGNRHWVTLVIVHQNGNYYGYYADSLGPDSGIDNNIRGALRECDINDDNVHNISVHQQ
CidB_IV(a/2)      INVGNRHWVTLVIVHQNGNYYGYYADSLGPDSGIDNNIRGALRECDINDDNVHNISVHQQ
CidB_IV(b/1)      INVGNRHWVTLVIVHQNGNYYGYYADSLGPDSGIDNNIRGALRECDINDDNVHNISVHQQ
CidB_IV(b/2)      INVGNRHWVTLVIVHQNGNYYGYYADSLGPDSGIDNNIRGALRECDINDDNVHNISVHQQ


CidB_I(a/1)       TDGHNCGIWVYENARDINQAIDQALQGNNNFGEKGEGIIGYIRGLLSAGIGNDTRQPRRN
CidB_I(a/2)       TDGHNCGIWVYENARDINQAIDQALQGNNNFGEKGEGIIGYIRGLLSAGIGNDTRQPRRN
CidB_I(b/1)       TDGHNCGIWVYENARDINQAIDQALQGNNNFGEKGEGIIGYIRGLLSAGIGNDTRQPRRN
CidB_I(b/2)       TDGHNCGIWVYENARDINQAIDQALQGNNNFGEKGEGIIGYIRGLLSAGIGNDTRQPRRN
CidB_II(a/2)      TDGHNCGIWVYENARDINQAIDQALQGNNNFGEKGEGIIGYIRGLLSAGIGNDTRQPRRN
CidB_II(a/1)      TDGHNCGIWVYENARDINQAIDQALQGNNNFGEKGEGIIGYIRGLLSAGIGNDTRQPRRN
CidB_III(c/1)     TDGHNCGIWVYENARDINQAIDQALQGNNNFGEKGEGIIGYIRGLLSAGIGNDTRQPRRN
CidB_III(a/1)     TDGHNCGIWVYENARDINQAIDQALQGNNNFGEKGEGIIGYIRGLLSAGIGNDTRQPRRN
CidB_III(b/1)     TDGHNCGIWVYENARDINQAIDQALQGNNNFGEKGEGIIGYIRGLLSAGIGNDTRQPRRN
CidB_IV(a/1)      TDGHNCGIWVYENARDINQAIDQALQGNNNFGEKGEGIIGYIRGLLSAGIGNDTRQPRRN
CidB_IV(a/2)      TDGHNCGIWVYENARDINQAIDQALQGNNNFGEKGEGIIGYIRGLLSAGIGNDTRQPRRN
CidB_IV(b/1)      TDGHNCGIWVYENARDINQAIDQALQGNNNFGEKGEGIIGYIRGLLSAGIGNDTRQPRRN
CidB_IV(b/2)      TDGHNCGIWVYENARDINQAIDQALQGNNNFGEKGEGIIGYIRGLLSAGIGNDTRQPRRN


CidB_I(a/1)       EQYFEDRRRDISQLLQNDPNLPSRRSDLIQAHPGIQHEIDPLLLQFLGLQYPQRGGGGAL
CidB_I(a/2)       EQYFEDRRRDISQLLQNDPNLPSRRSDLIQAHPGIQHEIDPLLLQFLGLQYPQRGGGGAL
CidB_I(b/1)       EQYFEDRRRDISQLLQNDPNLPSRRSDLIQAHPGIQHEIDPLLLQFLGLQYPQRGGGGAL
CidB_I(b/2)       EQYFEDRRRDISQLLQNDPNLPSRRSDLIQAHPGIQHEIDPLLLQFLGLQYPQRGGGGAL
CidB_II(a/2)      EQYFEDRRRDISQLLQNDPNLPSRRSDLIQAHPGIQHEIDPLLLQFLGLQYPQRGGGGAL
CidB_II(a/1)      EQYFEDRRRDISQLLQNDPNLPSRRSDLIQAHPGIQHEIDPLLLQFLGLQYPQRGGGGAL
CidB_III(c/1)     EQYFEDRRRDISQLLQNDPNLPSRRSDLIQAHPGIQHEIDPLLLQFLGLQYPQRGGGGAL
CidB_III(a/1)     EQYFEDRRRDISQLLQNDPNLPSRRSDLIQAHPGIQHEIDPLLLQFLGLQYPQRGGGGAL
CidB_III(b/1)     EQYFEDRRRDISQLLQNDPNLPSRRSDLIQAHPGIQHEIDPLLLQFLGLQYPQRGGGGAL
CidB_IV(a/1)      EQYFEDRRRDISQLLQNDPNLPSRRSDLIQAHPGIQHEIDPLLLQFLGLQYPQRGGGGAL
CidB_IV(a/2)      EQYFEDRRRDISQLLQNDPNLPSRRSDLIQAHPGIQHEIDPLLLQFLGLQYPQRGGGGAL
CidB_IV(b/1)      EQYFEDRRRDISQLLQNDPNLPSRRSDLIQAHPGIQHEIDPLLLQFLGLQYPQRGGGGAL
CidB_IV(b/2)      EQYFEDRRRDISQLLQNDPNLPSRRSDLIQAHPGIQHEIDPLLLQFLGLQYPQRGGGGAL


CidB_I(a/1)       QLGGERVISIDFGNPQSALDKIDGVSRVYNHSNSRGSR*
CidB_I(a/2)       QLGGERVISIDFGNPQSALDKIDGVSRVYNHSNSRGSR*
CidB_I(b/1)       QLGGERVISIDFGNPQSALDKIDGVSRVYNHSNSRGSR*
CidB_I(b/2)       QLGGERVISIDFGNPQSALDKIDGVSRVYNHSNSRGSR*
CidB_II(a/2)      QLGGERVISIDFGNPQSALDKIDGVSRVYNHSNSRGSR*
CidB_II(a/1)      QLGGERVISIDFGNPQSALDKIDGVSRVYNHSNSRGSR*
CidB_III(c/1)     QLGGERVISIDFGNPQSALDKIDGVSRVYNHSNSRGSR*
CidB_III(a/1)     QLGGERVISIDFGNPQSALDKIDGVSRVYNHSNSRGSR*
CidB_III(b/1)     QLGGERVISIDFGNPQSALDKIDGVSRVYNHSNSRGSR*
CidB_IV(a/1)      QLGGERVISIDFGNPQSALDKIDGVSRVYNHSNSRGSR*
CidB_IV(a/2)      QLGGERVISIDFGNPQSALDKIDGVSRVYNHSNSRGSR*
CidB_IV(b/1)      QLGGERVISIDFGNPQSALDKIDGVSRVYNHSNSRGSR*
CidB_IV(b/2)      QLGGERVISIDFGNPQSALDKIDGVSRVYNHSNSRGSR*
```

Supplementary Figure 3 | Complete sequences of the CidB variants detected in the different *w*Pip group of *Wolbachia* strains studied. Polymorphic regions represented by colored blocks in Figure 4 are reported on the alignment. The deubiquitylating (DUB) domain is underlined in red.

Supplementary Table 1 | Information on the four reference lines used for crossing type determination

Line Name	*Wolbachia* group	*Culex pipiens* subspecies	Country	Years of collection	Reference
Tunis	*w*Pip-I	*pipiens*	Tunisia	1992	Duron *et al.* 2005
Lavar	*w*Pip-II	*pipiens*	France	2003	Duron *et al.* 2005
Maclo	*w*Pip-III	*quinquefasciatus*	USA	1984	Duron *et al.* 2005
Istanbul	*w*Pip-IV	*pipiens*	Turkey	2003	Duron *et al.* 2005

Supplementary Table 2 | List of the primers used in this study.

Primers' number	Primers' name	Primers' sequences	PCR primers couple	Fragment size (bp)	Tm (°C)
1	wpip_0282_287981_dir	TGGTCAGGTGTAAGGTTGGA	1/2	1300	57
2	wpip_0282_289280_rev	CGACCAGAAACACCAAGAGT			
3	wpip0282_287607_Dir	ACTCAAACTACTGGTCTTTCTGT	3/4	81	59
4	wpip0282_288423_Rev	TGAACGCGAGAAAGAGCAAG			
5	wpip0282_289157_Dir	GATAGTCCGCAGTCTGAGCT	5/6	486	57
6	wpip0283_289642_Rev	ACCCATCACTATTAGGACGATCA			
7	wpip_283_289555_Dir	ACGGCAAACCTAAAAGTTGCT	7/8	1264	59
8	wpip_283_290818_Rev	TGATGGCTCCTTGTTGTTGC			
9	wpip0283_290614_Dir	AACTGGTTTTGCTTTTGGGAA	9/10	1280	57
10	wpip0283_291893_Rev	ATCTCACGAGCGTTGTTTCT			
11	wpip0283_291699_Dir	AGCACCAGGACAAAATTCGA	11/12	1211	59
12	wpip0283_292909_Rev	GCAGAAACAACCTAGAAGACCG			
13	wpip_0282_288758_rev	ACAAGCTACCACATTCCCAA	1/13	778	57
14	wpip_0282_QPCR_2_Dir	AGGTCCTGTATTTGATTTCTGGA	14/15	189	58
15	wpip_0282_QPCR_2_Rev	TGAACGCGAGAAAGAGCAAG			
16	wpip_0283_QPCR_1_Dir	TGAGTGTTTGGAGAATGAAGGA	16/17	135	58
17	wpip_0283_QPCR_1_Rev	TTCCCAAAAGCAAAACCAGTT			
18	wolpipdir	AGAATTGACGGCATTGAATA	18/19	151	58
19	wolpiprev	CGTCGTTTTTGTTTAGTTGTG			

Supplementary Table 3 | Accession numbers. Accession numbers for *cidA cidB* variants analyzed in Figure 3,4,5, 6,7, 8; Supplementary Figure 2 and 3

Gene name	Variant sequence submission name	Accession number	Gene name	Variant sequence submission name	Accession number
**cidA**	cidA_I(alpha/1)	MF444963	**cidB**	cidB_I(a/1)	MF444982
	cidA_I(gamma/1)	MF444964		cidB_I(a/2)	MF444983
	cidA_I(gamma/2)	MF444965		cidB_I(b/1)	MF444984
	cidA_I(beta/2)	MF444966		cidB_I(b/2)	MF444985
	cidA_II(alpha/1)	MF444967		cidB_II(a/2)	MF444986
	cidA_II(alpha/2)	MF444968		cidB_II(a/1)	MF444987
	cidA_II(beta/2)	MF444969		cidB_III(c/1)	MF444988
	cidA_III(alpha/1)	MF444970		cidB_III(a/1)	MF444989
	cidA_III(beta/2)	MF444971		cidB_III(b/1)	MF444990
	cidA_III(beta/1)	MF444972		cidB_IV(a/1)	MF444991
	cidA_III(beta/3)	MF444973		cidB_IV(a/2)	MF444992
	cidA_IV(alpha/1)	MF444974		cidB_IV(b/1)	MF444993
	cidA_IV(alpha/2)	MF444975		cidB_IV(b/2)	MF444994
	cidA_IV(gamma/1)	MF444976		cidB_IV(a/3)	MF444995
	cidA_IV(gamma/2)	MF444977		cidB_IV(b/3)	MF444996
	cidA_IV(delta/1)	MF444978			
	cidA_IV(delta/2)	MF444979			
	cidA_IV(beta/1)	MF444980			
	cidA_IV(beta/2)	MF444981			
