# Supplementary material for: Culex pipiens crossing type diversity is governed by an amplified and polymorphic operon of Wolbachia
Source: Nat Commun. 2018 Jan 22;9:319. doi: 10.1038/s41467-017-02749-w (PMC5778026; doi:10.1038/s41467-017-02749-w)
Supplement: Supplementary file 3 — Description of Additional Supplementary Files [file 41467_2017_2749_MOESM3_ESM.pdf]

File Name: Supplementary Data 1

Description: Detection of the *cidA*_IV (δ) and *cidB*_IV (a/2) variants with PCR-RFLP tests specific in 180 *C. pipiens* isofemale lines.
