# Supplementary material for: Author Correction: Culex pipiens crossing type diversity is governed by an amplified and polymorphic operon of Wolbachia
Source: Nat Commun. 2018 Apr 11;9:1491. doi: 10.1038/s41467-018-03799-4 (PMC5895800; doi:10.1038/s41467-018-03799-4)

Supplementary Figure 1. Unprocessed gel image of double digestion with Apo1 and Hpy188I of a 778 bp fragment of *cidA* gene which was amplified with primers 1/13 related to Figure 7c.

a. Unprocessed gel image of the left electrophoresis gel of the Figure 7c.

b. Unprocessed gel image of the right electrophoresis gel of the Figure 7c.

a

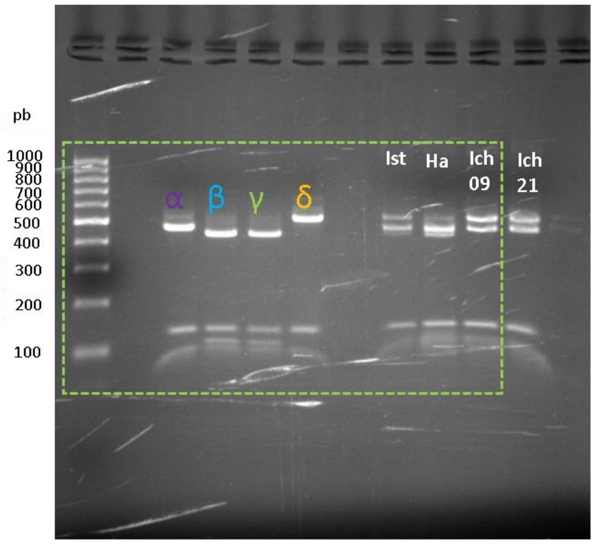

b

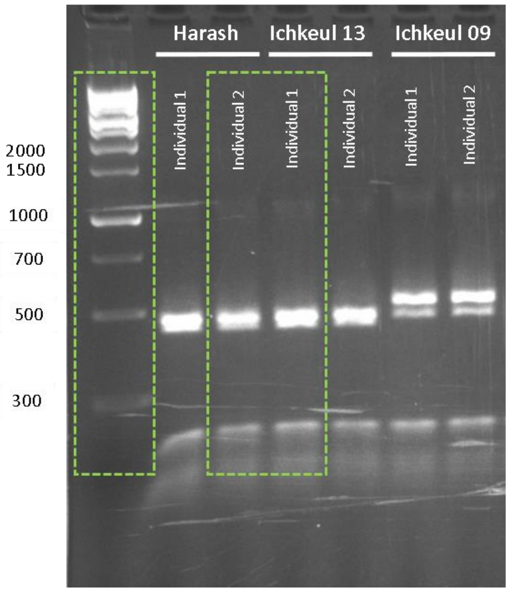

Supplementary Figure 2. Unprocessed gel image of double digestion with Apo1 and Hpy188I of a 778 bp fragment of *cidA* with the line Istanbul, Harash, Ichkeul 13 and Ichkeul 09 ran on the same electrophoresis gel related to Figure 7c.

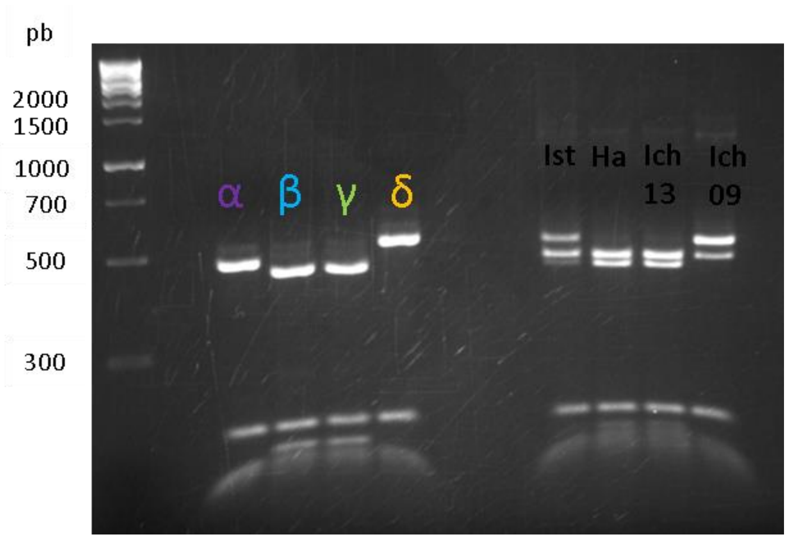

Supplementary Figure 3. Unprocessed gel image of double digestion with Taqa1 and Ban1 of a 1267–1276 bp fragment of *cidB* gene which was amplified with primers 7/8 related to Figure 8c.

a. Unprocessed gel image of the left electrophoresis gel of the Figure 8c.

b. Unprocessed gel image of the right electrophoresis gel of the Figure 8c.

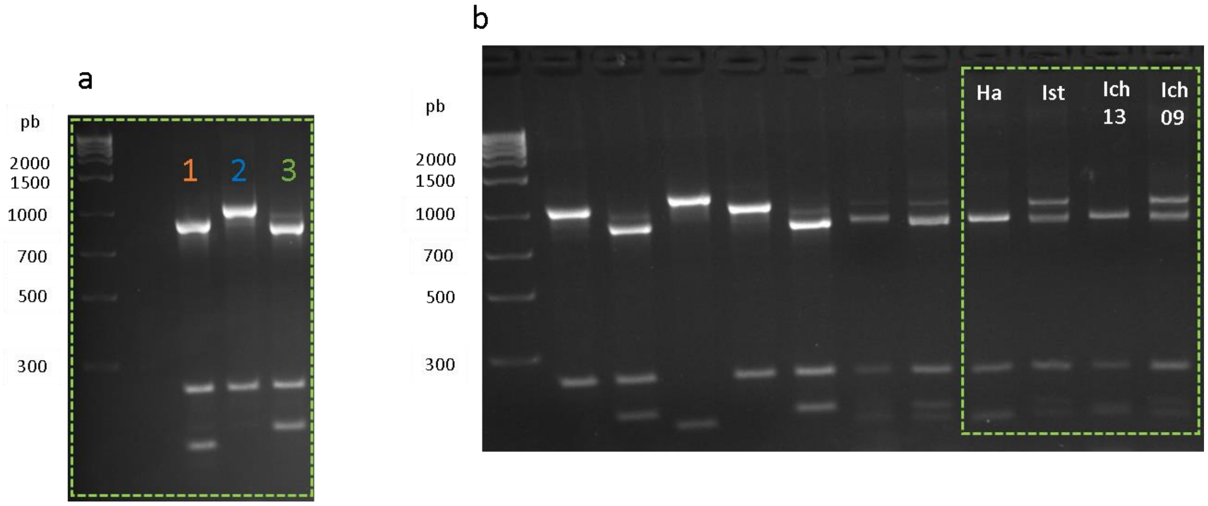

Supplement: Supplementary file 1 — Supplementary Information [file 41467_2018_3799_MOESM1_ESM.pdf]
